# High burden of undernutrition among primary school-aged children and its determinant factors in Ethiopia; a systematic review and meta-analysis

**DOI:** 10.1186/s13052-020-00881-w

**Published:** 2020-08-26

**Authors:** Moges Agazhe Assemie, Alehegn Aderaw Alamneh, Daniel Bekele Ketema, Ali Mekonen Adem, Melaku Desita, Pammla Petrucka, Mekdes Marew Ambaw

**Affiliations:** 1 Biostatstics Unit, Department of Public Health, College of Health Science, Debre Markos University, P.O. Box: 269, Debre Markos, Ethiopia; 2 Department of Human Nutrition and Food Sciences, College of Health Sciences, Debre Markos University, Debre Markos, Ethiopia; 3 Biostatstics Unit, College of Health Science, Assossa University, Assossa, Ethiopia; 4 Department of Midwifery, College of Health Sciences, Debre Markos University, Debre Markos, Ethiopia; 5 College of Nursing, University of Saskatchewan, Saskatoon, Canada; 6 School of Life Sciences and Bioengineering, Nelson Mandela African Institute of Science and Technology, Arusha, Tanzania; 7 Department of Agricultural Economics, College of Agriculture and Resource Management, Dilla University, Dilla, Ethiopia

**Keywords:** Undernutrition, Primary school-aged children, Stunting, Underweight, Thinness/wasting, Ethiopia

## Abstract

**Background:**

Undernutrition remains a major public health concern affecting both children and adolescents in Ethiopia. However, little attention has been given to the undernutrition of primary school-aged children, with their exclusion within national surveys. Therefore, this systematic review and meta-analysis was conducted to determine pooled estimate and determinant factors of undernutrition among primary school-aged children (6 to 15 years of age) in Ethiopia.

**Method:**

We systematically retrieved available articles on the prevalence of undernutrition in primary school-aged children in Ethiopia by using a number of computerized databases, including PubMed, Scopus, Cochrane Library, Google Scholar, and Science Direct between September 1 and November 25, 2019. Two authors independently extracted relevant data using a standardized data extraction form. Heterogeneity among included studies was assessed with the Cochrane Q test statistics and Higgins *I*^*2*^ tests. The pooled estimates and determinant factors of school-aged undernutrition were assessed with random-effects model using Stata/se Version 14.

**Result:**

We have retrieved 30 eligible articles with pooled sample size of 16,642 primary school- aged children to determine the prevalence of undernutrition in Ethiopia. Hence, the pooled prevalence of stunting, underweight, and wasting were found to be 21.3% (95% CI: 17.0, 25.5), 18.2% (95% CI: 14.4, 22.0) and 17.7% (95% CI, 13.5, 21.8) respectively. Heterogeneity was assessed by doing subgroup analysis for study province/region. Thus, the highest prevalence of stunting was 27.6% (95% CI, 20.7, 34.5) and underweight 22.7% (95% CI, 19.2, 26.3) in Amhara Region while, in the instance of wasting, it was 19.3%(95% CI: 5.1, 33.4) in Southern Nations, Nationalities and People’s Region. Maternal educational status (OR = 1.91, 95% CI: 1.33, 2.73), age of school-aged child (OR = 0.56, 95% CI: 0.44, 0.72) and sex of school-aged child (OR = 0.73, 95% CI: 0.62, 0.85) were found to be significantly associated with stunting. Maternal educational status (OR = 0.6, 95% CI: 0.36, 0.9) and age of school-aged child (OR = 2.74, 95% CI: 1.81, 4.14) were associated with thinness/wasting. Parasitic infection (OR = 2.02, 95% CI: 1.10, 3.73) were associated with underweight of school age children.

**Conclusion:**

The prevalence of stunting and underweight among primary school-aged children are moderately high while acute undernutrition (wasting) is more critical than under-five national average as reported in the 2016 Ethiopian Demography and Health Survey. Therefore, this finding warrants the need to design a school-aged children nutrition survey and expand school feeding programs to improve the nutritional status of primary school-aged children in the country. In addition, emphasis should be given to female school-aged children in the early school years, creating awareness for those mothers who lack formal education, and preventing and treating/deworming parasitic infection. Moreover, researchers must conduct research in province/regions which have not yet studied school aged children’s nutritional status to date.

## Background

Child nutrition is an index and prerequisite for the national investment in the development of a nation’s future social capital; therefore, ensuring optimal child growth and development is important to accelerate economic development of nations [1, 2]. Undernutrition continues to be a primary cause of disease susceptibility, morbidity, and mortality among school-aged children particularly in resource limited countries which globally accounts for half of all deaths in this cohort [3, 4]. Undernutrition is caused by a lack or imbalance of nutrients in the body. It includes stunting, wasting, and underweight which is a consequence of consuming too few essential nutrients or excreting them more rapidly than they can be replaced [5].

Globally, more than 200 million school-aged children are stunted and underweight which contributes to 2.2 million deaths annually [4]. If an interventional action is not taken, the cognitive and physical impairment will increase to one billion by 2020 [6–8]. Like under-five children, school-aged children are among the most vulnerable segments of the population to undernutrition in Sub-Saharan Africa [6, 9]. Undernutrition among school-aged children results in poor cognitive development, lower school performance, reduced body size, reduced muscular strength, and depleted work capacity [8, 10–13].

Primary school-aged children who are undernourished can exhibit improvement in their growth and development if their environment improves [14–16]. Hence, special attention to proper nutritional surveys or suitable nutritional intervention programmes in a community can provide the baseline upon which to assess the occurrence of undernutrition among primary school-aged children [17]. Therefore, targeted interventions in primary school-aged children can help to complement efforts in the preschool years and can reduce the perpetuation of undernutrition and its effects on health [12].

Therefore, we conducted this systematic review and meta-analysis to bridge the data inconsistencies and gaps in the existing evidence regarding nationally representative undernutrition among primary school-aged children in Ethiopia. This finding will help decision makers and other concerned stakeholders undertake an intensified effort to design a school-centric survey and implement effective and efficient interventions to decrease undernutrition among primary school-aged children.

## Methods

### Source of evidence

Systematic review and meta-analysis from computerized databases and gray literatures form the evidentiary bases of this study.

### Searching strategies and data selection

Preferred reporting items for systematic review and meta-analysis (PRISMA) guidelines were used to prepare and present this systematic reviews and meta-analysis [18]. To identify relevant articles, a comprehensive search between 2006 and September 3, 2019 was performed in the databases of PubMed, Science Direct, Google Scholar, and Cochrane Library. All searches were limited to articles written in English and grey literature was searched through the review of reference lists and hand searches. Moreover, to find unpublished papers, select research centers, including Addis Ababa Digital Library and Saint Mary’s University repositories were searched. Studies identified by our search strategy were retrieved and managed using Endnote X7 (Thomson Reuters, Philadelphia, PA, USA) software. The search was conducted from September 1 to November 25, 2019. During the search process the authors used the following keywords and MeSH terms: prevalence AND undernutrition OR stunting OR wasting OR underweight OR thinness AND associated factor OR determinant factor AND primary school-aged children AND Ethiopia.

### Study population

#### Inclusion criteria

##### Study area

All studies conducted in Ethiopia.

##### Study design

All observational study designs reporting the prevalence of undernutrition.

##### Population

**S**tudies involving primary school-aged children (6–15 years).

##### Language

Only articles reported in English language.

##### Publication condition

Published and unpublished articles.

##### Exclusion criteria

Articles, which were not fully accessible after two-email contacts, were excluded because of inability to assess the quality of articles in the absence of full text.

### Data extraction

All relevant articles were screened and extracted by two authors (AA and MM) independently using a standardized data extraction format in Microsoft™ Excel tool adapted from the Joanna Briggs Institute (JBI) [19]. Name of first authors, publication year, study area, study province, study design, mean age, response rate, sample size, and 95% CI prevalence of undernutrition as (stunting, underweight and thinness/wasting) were extracted from all selected full papers.

### Quality assessment

In this meta-analysis, the qualities of the included article were assessed using a modified version of the Newcastle-Ottawa Scale for cross-sectional studies adopted from Madhavan et al. [20]. This tool has three main sections. The first section, scored on the basis of one to five stars, focuses on methodological quality of each study (i.e., sample size, response rate, and sampling technique). The second section of the tool considers the comparability of the study cases with a possibility of two stars to be gained. The last section is concerned with the outcomes and statistical analysis of the original study with a possibility of three stars to be gained. In addition, quality appraisal of included studies was evaluated by two authors (MD and MM) independently and any discrepancy was resolved by a third author (MA). Articles attaining a Newcastle-Ottawa Scale (NOS) score of ≥7 stars out of 10 were considered as high quality and eligible for inclusion. In this study, all eligible studies were determined to have high quality scores.

### Risk of bias assessment

Two authors (DB and MA) assessed the risk of bias for included articles independently by using the Hoy et al.’s internal and external validity tool for prevalence studies based on a review of the literature [21]. The tool consists of ten items addressing four domains of bias plus a summary risk of bias assessment [21]. Any scoring difference was resolved by discussion mediated by a third author (AM).

### Data collection tool used by included studies

All included studies used a structured pre-tested questionnaire for face to face interviews and anthropometric measurement for weight was measured with minimal clothing and no shoes using an electronic beam balance in kilogram (kg) to the nearest of 0.1 kg and length was measured with standing position in centimeters (cm) to the nearest of 1 cm. Finally the anthropometric measures of weight, height, and age values were converted into Z-scores of the indices according to WHO standards [22]. Since the tools used by primary studies are pre-tested and standard measurements were used at various settings, it permits meaningful comparisons.

### Outcome assessment

There were two outcomes considered for this systematic review and meta-analysis. The first outcome was to determine the pooled prevalence of undernutrition in terms of stunting, underweight and thinning/wasting among primary school-aged children in Ethiopia. Underweight is reflected when the weight for age is more than 2 standard deviations (SD) below the WHO Child Growth Standards median; stunting reported when height for age is more than 2 SD below of the WHO Child Growth Standards median; and wasting is weight for height is more than 2 SD below the WHO Child Growth Standards median [23].

The second outcome was to identify determinant factors of undernutrition in the form of the log odds ratio. For each factor, the odds ratio was calculated based on the binary outcome data reported by each study. If the factors were not reported in sufficient details across studies (less than two studies), the log odds ratios were not pooled. A narrative synthesis for the reports of systematic reviews was done for quantitative data as forest plots without pooled estimates, which were not appropriate for meta-analysis due to low number. The factors assessed for this review was educational status of mothers (illiterate versus literate), sex of the child (female versus male), age of children (5–10 years versus 10–15), family size (< 5 versus > 5), frequency of meals per day (< 3 versus > 3), infection (yes versus no), source of water (protected versus unprotected), hand washing with soap (yes versus no), and availability of latrine (yes versus no).

### Data processing and analysis

A Microsoft™ Excel spreadsheet form was used to present the extracted data from the primary study and further analysis was done by STATA/se™ Version 14 statistical software. The standard error was calculated based on binomial distribution. Heterogeneity was assessed by computing *p*-values of Higgins’s *I*^2^ test statics and Q-statistics among reported prevalence. Higgins’s I^2^ statistic measures the difference between sample estimates (in percentage) which is due to heterogeneity rather than to sampling error. The pooled effect was estimated with a random effects meta-analysis model. Subgroup analysis was performed based on study province, sample size, and publication year to minimize random variations between the estimates of the primary study. In addition, univariate meta-regression was computed to identify the possible sources of heterogeneity by considering sample size, quality score, and publication year. Publication bias was assessed visually by funnel plot and statistically supported with confirmatory and/or objectivity testing with Egger’s and Begg’s test at a 5% level of significance [24]. Point prevalence, as well as 95% confidence intervals, was presented in a forest plot format. In this plot, the size of each box indicated the weight of the study, while each crossed line referred to a 95% confidence interval with the mean effect at the center. The determinant factors of undernutrition were determined by a log odds ratio at 95% level of significance.

## Results

**Fig. 1:**
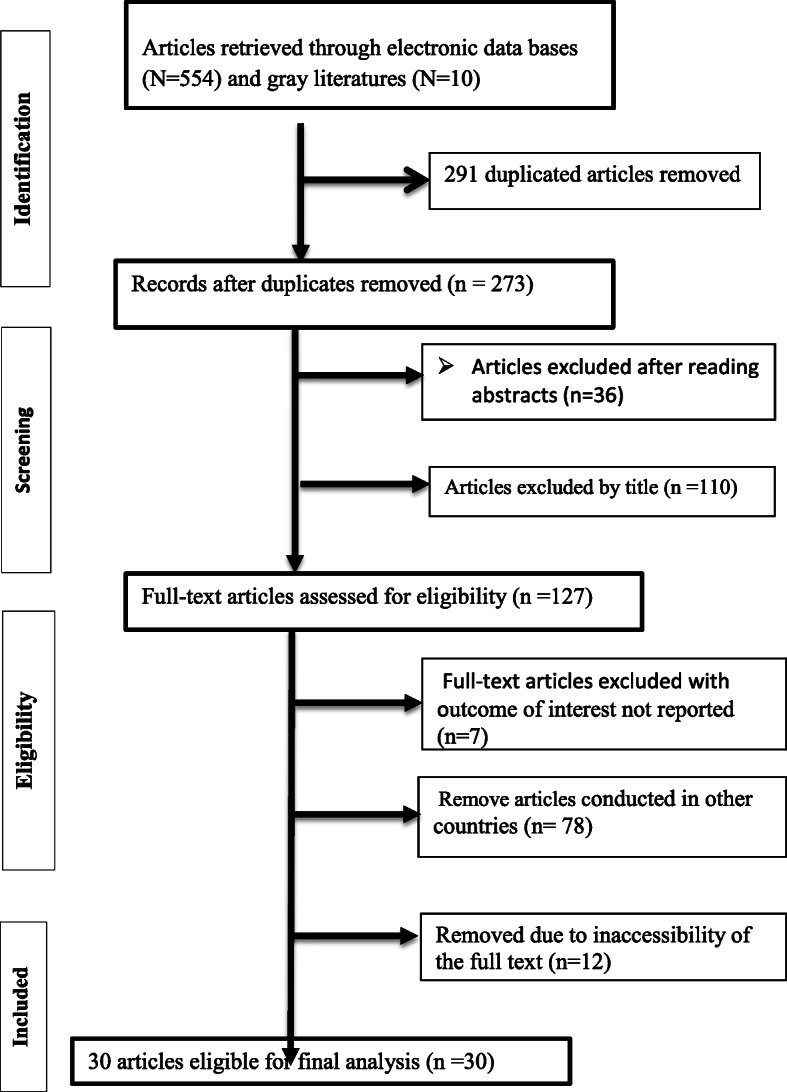
Flow chart of selecting articles for systematic review and meta-analysis to determine undernutrition among primary school-aged children in Ethiopia

First, 564 studies reported the prevalence of undernutrition and associated factors from the computerized database search. We then excluded 291 articles due to duplication and the remaining 273 studies were assessed for eligibility. A further 146 articles were removed after reading title and abstract. Finally, 30 of 127 articles were found eligible, accessible, and were included for final meta-analysis after reading the full text studies (Fig. 1).

### Description of included primary studies

As described in Table 1, thirty cross-sectional articles published between 2006 and 2019 were including with a total sample size of 16,642 and a response rate of 97.4%. The smallest sample size was 97 in a study in Gondar, Amhara [32] while the largest sample size was 2241 in a study done at Bahir Dar and Mecha, Amhara [54].

**Table 1:** Descriptive summary of 30 articles eligible in the meta-analysis of undernutrition among primary school children in Ethiopia between 2006 and September 2019

Authors	Publication/year	Region	Study area	Sample	Response	Undernutrition (%)		
						Stunting	underweight	wasting
Zerfu et al. [25]	2006	Addis Ababa	Addis Ababa	1208	100	24	NA	NA
Worku et al. [26]	2009	Amhara	Gondar	322	100	27	34.8	50
Reji et al. [27]	2011	Oromia	Adama	358	99.7	12.6	7.2	NA
Nguyen et al. [28]	2012	Amhara	Angolela	664	95.5	11	20.8	19.6
Amare et al. [29]	2012	Amhara	Gondar	100	97	23	11	18
Amare et al. [30]	2013	Amhara	Gondar	405	100	25	15	8.9
Mahmud et al. [31]	2013	Tigray	Mekelle	600	97.2	35	NA	34
Kidane et al. [32]	2014	Tigray	Wukero	384	100	9.4	15.4	9.9
Mekonnen et al. [33]	2014	Oromia	Fincha	458	98.9	11.5	NA	13.2
Damie et al. [34]	2015	Oromia	Chiro	319	91.2	7.2	24.4	NA
Wolde M et al. [35]	2015	SNNP	Dale	450	98.8	10.3	19	14
Alelign et al. [36]	2015	Amhara	Durbet	403	95.3	11.2	27.1	NA
Degarege et al. [37]	2015	Addis Ababa	Addis Ababa	459	100	19.6	15.9	NA
Begna et al. [38]	2016	SNNP	Delo-mena	518	95	4.5	13.6	17.1
Zerdo et al. [39]	2017	SNNP	Chencha	406	100	8.9	4.2	NA
Tefera et al. [40]	2017	Harerie	Babile	632	100	12.8	NA	18.1
Birmeka et al. [41]	2017	SNNP	Enemorena	641	100	39	11.8	40.9
Wordofa et al. [42]	2017	Oromia	Sululeta	510	100	10.8	NA	NA
Molla et al. [43]	2018	SNNP	Yirgacheffe	448	98.9	10.8	12.9	5.2
Menber et al. [44]	2018	Amhara	Haik	388	93.7	11.3	NA	NA
Asmare et al. [45]	2018	Amhara	Markos	436	98.6	27.5	20.4	8.5
Hailegebriel et al. [46]	2018	Amhara	Bair Dar	422	90.5	41.6	25.9	26.7
Tariku et al. [47]	2018	Amhara	Arba Minch	389	96	41.9	NA	8
Enyew et al. [48]	2018	Amhara	DebreTabou	399	100	32.4	24.3	NA
Lisanu et al. [49]	2018	Amhara	Mecha	802	99.8	37.9	NA	NA
Wolde et al. [50]	2019	SNNP	Meskan	408	93	16.9	NA	NA
Belay et al. [51]	2019	Amhara	Armachiho	848	97.6	35.5	NA	9.9
Getaneh et al. [52]	2019	Amhara	Gondar	523	96.5	46.5	NA	8.8
Bantie et al. [53]	2019	Amhara	BDR	370	98.6	15.1	NA	NA
Felek et al. [54]	2016	Amhara	Mecha	2372	94.5	NA	24.4	10.8

Regarding the prevalence of undernutrition, the smallest prevalence of stunting was 4.5% [38] while the largest was 46.5% [52]. The prevalence of underweight ranged from 4.2% [39] to 34.8% [26]; whereas wasting (thinness) ranged between 5.2% [43] to 50% [26].

### Meta-analysis of undernutrition among primary school children in Ethiopia

**Fig. 2:**
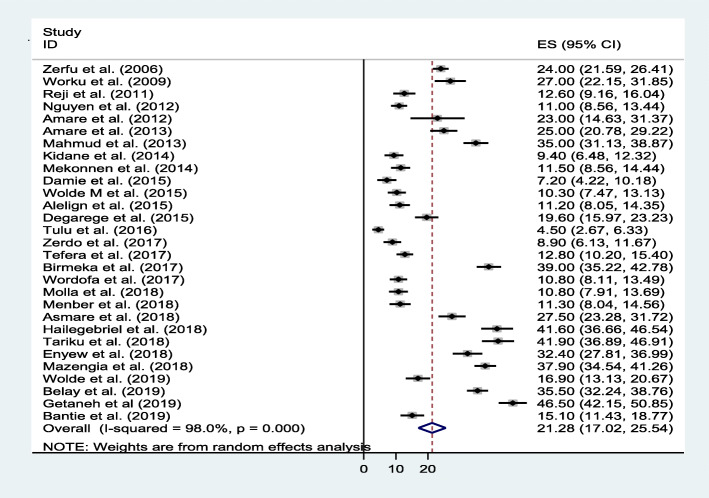
Forest plot of the pooled prevalence of stunting among school-aged children in Ethiopia

Thirty articles with a pooled sample size of 16,642 school-aged children and response rate of 97.4% were considered in this systematic review and meta-analysis. The pool prevalence of stunting, underweight, and wasting in Ethiopia were 21.3% (95% CI: 17.0, 25.5), 18.2% (95% CI: 14.4, 22.0), and 17.7% (95% CI: 13.5, 21.8) respectively (Figs. 2, 7 & 8).

### Stunting

The estimated prevalence of stunting among school-aged children in Ethiopia from 29 articles was 21.3%. There was high heterogeneity found within the included articles as revealed in forest plot (I2 = 98%, *p* < 0.001) (Fig. 2).

Thus, the subgroup analysis in the study province provide the highest prevalence of stunting in Amhara region 27.6% (95% CI: 20.7, 34.5)(I^2^ = 97.8, *p <* 0.001) followed by Southern Nations, Nationalities and People’s (SNNP) Region 15.0% (95% CI: 26.5, 23.4)(I^2^ = 98.2, *p <* 0.001) and the lowest was noted in Oromia Region 10.5% (95% CI: 8.2, 12.7)(I^2^ = 54.8, *p* = 0.084) (Table 2).

**Table 2:** Subgroup analysis of primary school children undernutrition (stunting, underweight and wasting) by study province/region of Ethiopia between 2006 and September 2019

Category of undernutrition	Province/region	Study number	Sample size	Prevalence of undernutrition
Stunting	Amhara	14	6471	27.6 (20.7,34.5)
	SNNP	6	2871	15 (6.5,23.4)
	Oromia	4	1645	10.5 (8.2,12.7)
	Tigray	2	984	2202 (2.9,47.3)
	Addis Ababa	2	1667	22.0 (17.7,26.3)
	Hararie	1	632	12.8 (10.2,15.4)
Underweight	Amhara	10	5523	22.7 (19.2,26.3)
	SNNP	5	2463	12.2 (7.1,17.2)
	Oromia	2	677	15.7 (1.2,32.4)
	Tigray	1	384	15.4 (11.8,19.0)
	Addis Ababa	1	459	15.9 (12.6,19.3)
Wasting	Amhara	10	6481	16.5 (11.7,21.3)
	SNNP	4	2057	19.3 (5.1,33.4)
	Oromia	1	458	13.2 (10.1,16.3)
	Hararie	1	632	18.1 (15.121.1)

**Fig. 3:**
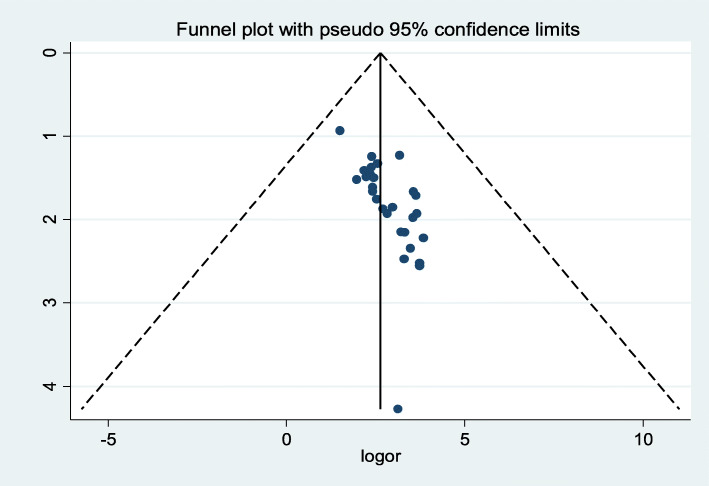
Funnel plot with 95% confidence limits of the pooled prevalence of stunting among school-aged children in Ethiopia

The presence of publication bias was assessed with subjectivity test of bias funnel plot visually and found substantially asymmetric and a further confirmatory test of objectivity to indicate publication bias was done with Egger’s test (*p-*value < 0.001) (Fig. 3). Even though the funnel plot showed substantial symmetry, the Egger’s test shows presence of bias. Thus, we performed a nonparametric trim and fill analysis to handle the observed publication bias for estimating the number of missing studies that might exist and in reducing and adjusting publication bias in meta-analysis.

### Factors associated with stunting in Ethiopia

In this systematic review and meta-analysis of stunting in Ethiopia, the associated factors affecting stunting were frequency of meals three times per day with odd ratio of 0.45(95%CI: 0.28, 0.72) and hand washing practice with odds of 0.73(95%CI: 0.48, 1.12) were reported from a single article as a result were not able to generate the pooled estimate. Family size of below five members was reported from three studies with pooled odd ratio of 7.95(95% CI: 0.62, 15.3), which was not found statistically significant.

**Fig. 4:**
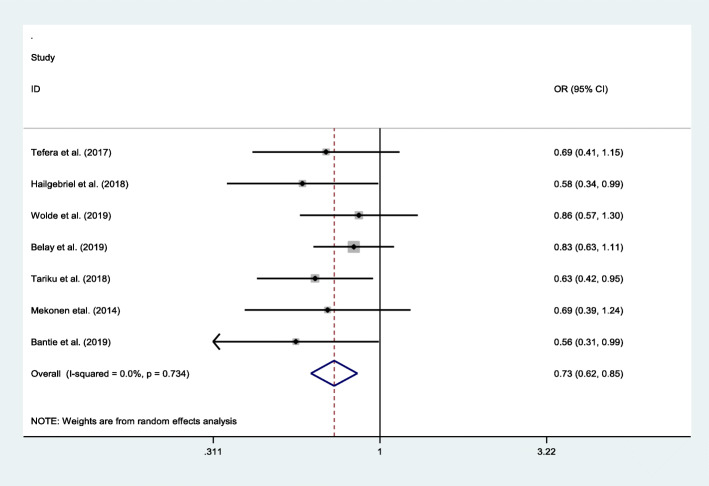
Pooled odd ratio between stunting and sex of school-aged children

Sex with pooled odd ratio of 0.73 (95% CI: 0.62, 0.85), age of children with pooled odd ratio of 0.56 (95% CI: 0.44, 0.72), and maternal education with pooled odd ratio of 1.91(95% CI: 1.33, 2.73) were found statistically significant. As we have examined the association between sex and stunting among school-aged children from seven [7] studies; the findings showed that sex had an association with prevalence of stunting, with the odds of stunting 27% higher among males as compared to females (OR = 0.73, 95% CI: 0.62, 0.85). The results did not indicate presence of heterogeneity (I2 = 0.0% and *p* = 0.734) (Fig. 4).

**Fig. 5:**
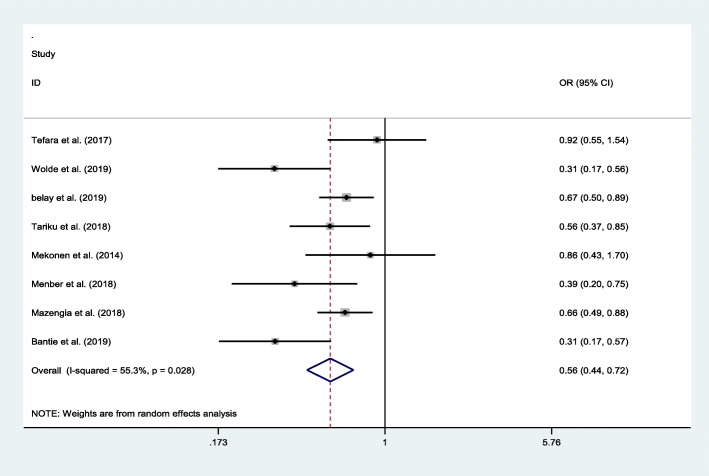
Pooled odd ratio between stunting and school-aged children

The association between age category and stunting was calculated from eight studies. Accordingly, the odds of stunting was 79% higher among school-aged children less than 10 years of age as compared to children 10–15 years old (OR = 0.56, 95% CI: 0.44, 0.72). The statistics showed moderate heterogeneity (I2 = 55.3% and *p* = 0.028) across the included studies (Fig. 5).

**Fig. 6:**
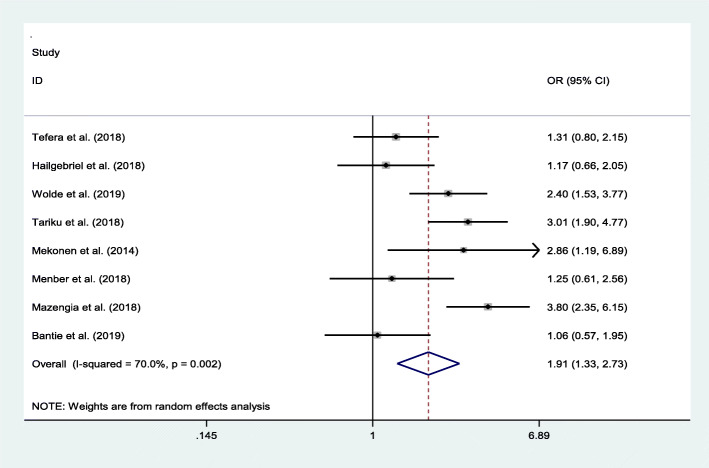
Pooled odd ratio between stunting and maternal educational status

We observed the association between maternal education and stunting from eight studies. The prevalence of stunting was 1.91 times higher among school-aged children whose mothers had no formal education in comparison to the more educated cohort (OR = 1.91, 95% CI: 1.33, 2.73). The resulting statistics showed moderate heterogeneity (I2 = 70% and *p* = 0.002) across the included studies (Fig. 6).

### Underweight

**Fig. 7:**
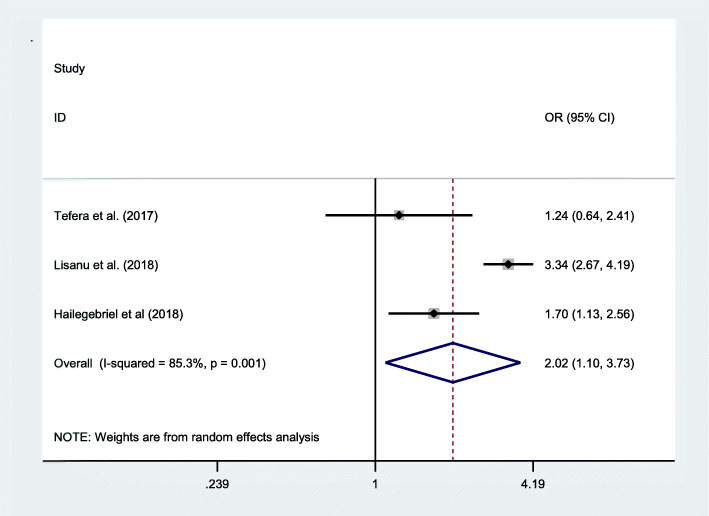
Forest plot of the pooled prevalence of underweight among school-aged children in Ethiopia

Prevalence of underweight in school-aged children in Ethiopia from 18 studies was 18.2%. A high heterogeneity was observed across the included articles as revealed in forest plot (I2 = 96.1%, *p <* 0.001) (Fig. 7).

Subsequently, subgroup analysis by the study province to indicate source of heterogeneity provides the highest prevalence of underweight at 22.7% (95% CI: 19.2, 26.3) (I^2^ = 88.1, *p <* 0.001) in Amhara region followed by 12.2% (95% CI: 7.1, 17.2) (I^2^ = 94.1, *p* < 0.001) in SNNP region (Table 2).

### Factors associated with underweight in Ethiopia

As we have studied the associated factors of underweight among school-aged children in Ethiopia, we had observed protected source of water and hand washing with soap were reported form only one article as associated factor with underweight with odd ratio of 1.18 (95% CI: 0.7, 1.98) and 2.62 (95% CI: 1.02, 6.72) respectively. Two factors (sex of school-aged children and educational status of mothers) were reported consistently over three different articles but both were not statistically associated with underweight (OR = 1.17, 95% CI: 0.54, 2.56) and (OR = 1.00, 95% CI: 0.44, 2.30) respectively.

**Fig. 8:**
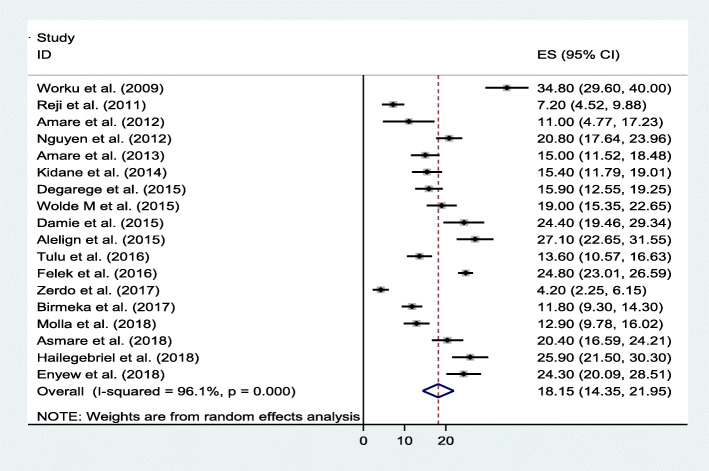
Pooled odd ratio between underweight and parasitic infection

The presence of parasitic infection reported from three studies with pooled odd ratio 2.02(95% CI: 1.10, 3.73) was found significantly significant. Thus, the prevalence of underweight was two times higher among those children infected with parasitic infection as compared to non-infected children (Fig. 8).

### Wasting

**Fig. 9:**
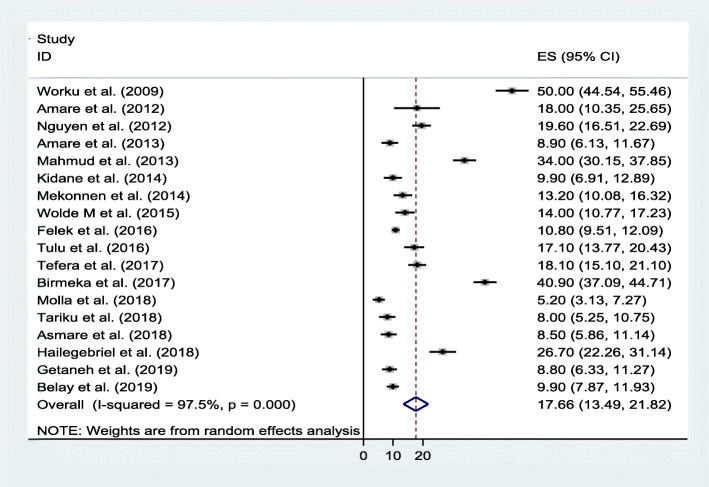
Forest plot of the pooled prevalence of wasting/thinness among school-aged children in Ethiopia

The pooled estimates of wasting in Ethiopia among school-aged children from 18 articles was 17.7% and the heterogeneity between the studies were observed across the included articles as revealed in forest plot (I2 = 97.5%, *p <* 0.001) (Fig. 9).

**Fig. 10:**
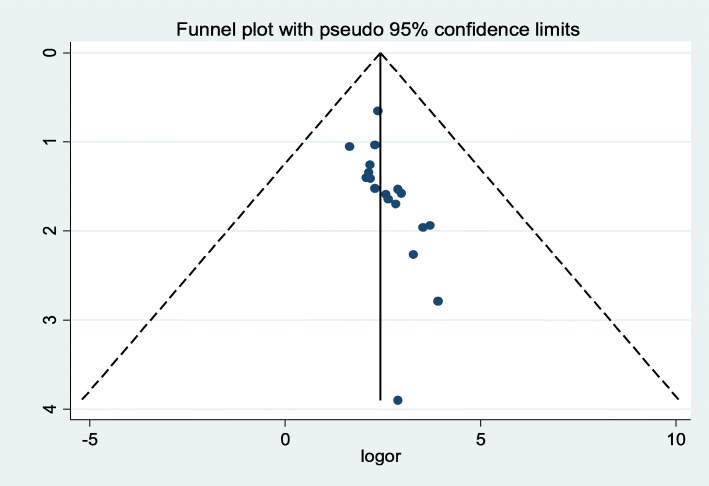
Funnel plot with 95% confidence limits of the pooled prevalence of thinness among school-aged children in Ethiopia

Accordingly, the subgroup analysis by study province to show source of heterogeneity of wasting among the included articles indicated the highest prevalence of wasting was observed at 19.3% (95% CI: 5.1, 33.4) (I^2^ = 98.9, *p <* 0.001) in SNNP followed by 16.5% (95% CI: 11.7, 21.3) (I^2^ = 96.9, *p <* 0.001) in Amhara region (Table 2). To determine publication bias, we performed funnel plot to show bias graphically, yielding moderate asymmetry. Further confirmation of bias was assessed with objectivity Egger’s test, the presence of publication bias was noted with (*p*-value =0.005) **(**Fig. 10**)**. A nonparametric trim and fill analysis was conducted to handle and adjusting the observed publication bias.

### Factors associated with wasting in Ethiopia

**Fig. 11:**
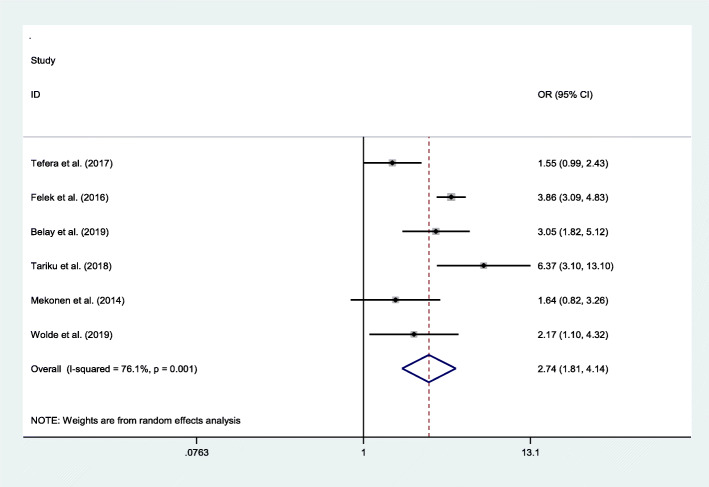
Pooled odds ratios between thinness and maternal educational status

The association between age category and wasting was calculated from six studies. The prevalence of wasting was 2.74 times higher among school-aged children 5 to10 years age as compared to those aged 10 to15 years (OR = 2.74, 95% CI: 1.81, 4.14) from a random effects model. The result of the statistics showed moderate heterogeneity (I2 = 76.1% and *p* = 0.001) across the studies (Fig. 11).

**Fig. 12:**
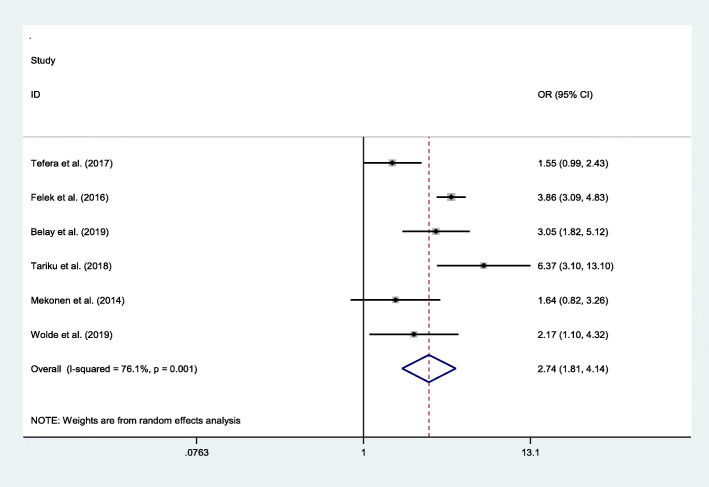
pooled odd ratio between thinness and school-aged children

In addition we have calculated the log odds ratio for educational status of mothers on wasting/thinness of school-aged children, finding that the odds of wasting is 1.66 times higher among children at the age of 5 to10 years as compared to 10 to15 years of age (OR = 0.6, 95% CI: 0.36, 0.9). Moderate heterogeneity was observed over the included studies with their respective log odds ratio (I2 = 76.1%, *p =* 0.001) (Fig. 12).

## Discussion

Primary school children are amongst the most vulnerable segments of the population for undernutrition, which is one of the key indicators for improving health, academic performance, and country development. Undernutrition among school-aged children is explained with the three common anthropometric indicators includes height-for-age (stunting), weight-for-age (underweight) and weight-for-height (thinness/wasting) [25]. Hence, the effect of undernutrition on child morbidity and mortality reduction can be determined by considering all undernutrition indicators through appropriate and standard anthropometric measurements [27] making this systematic review and meta-analysis important to show what is currently known about undernutrition among primary school children at the national level.

In our study, the prevalence of stunting, underweight, and wasting/thinness in Ethiopia were found to be 21.3, 18.2 and 17.7% respectively among primary school children. The magnitude of stunting in this study is relatively congruent with the studies conducted in west Bengal 23.0% [28] and Mexico 22.3% [29]; lower than findings from China (25.6%) [30], Indonesia (28.11%) [31] and Egypt (34.2%) [33], and higher than reports from Iraq (18.7%) [34], India (17%) [35] and Nigeria (17.4%) [36]. Underweight school-aged children in Ethiopia (18.2%) in this study showed lower levels from than reports from Bangladesh (43%) [37], Yemen (46.2%) [40], and Nepal (27.4%) [41] but higher than a report from Tanzania (5.7%) [42]. The third anthropometric indicators of undernutrition is wasting (thinness) which, in our study, had higher prevalence than the studies done in the western region of Nepal (12%) [44] and Burkina Faso (13.7%) [45]. In contrast, it is lower than a study from Ghana at 19.4% [46]. The possible explanation for these variations could be attributable to the difference in the parasitic infection, livelihood of the population economy, seasonal, cultural, socio-economical, and geographical diversity.

Parasitic infection is one of the determinate factors for undernutrition as supporting by the studies conducted in different parts of the world [47, 48]. This could be due to parasitic infection competing for the nutritional intake of children resulting in impairment of the immune systems and contributing to susceptibility to many diseases that extend its impact to underweight and cognitive impairment, decreased school attendance [49, 50].

Like the studies conducted in Bangladesh and India [37, 51], the current study found that the odds of undernutrition were higher in children below 10 years as compared to older ones which reflects the nature of stunting as a chronic nutritional problem. This could be due increased risk of intestinal parasitic infection among younger age hand less habit of not washing hands before eating among and cannot protect themselves from parasitic infection sources.

Being male was found to be a higher risk factor for malnutrition similar to previous work conducted in India [51]. This could be male children are usually more mobile and undertake different playing activities that make them loss grater energy from their body. On the other hand, females are usually give more attention to their personal hygiene than males and less mobile in their behavior and stay at home, have more access to different food staff at early age [53].

In addition, maternal educational status is a significant contributing factor for undernutrition. Similar to the studies in Indonesia and Nigeria [31, 36], the odds of undernutrition for primary school children were higher among mothers who had not formal education (illiterate) as compared to the literate cohort. This finding could be explained by educated mothers having better understanding of how to maintain the health of their child and their nutrition with the available resources. They can also contribute greater effort at home and can raise productivity and contributions of the women to the families’ economic situations, bring attention to improved child nutrition, and regulate the family size. As a result, education of girls is education of all and ultimately would be the wise method of reducing undernutrition.

## Limitation of the study

This systematic review and meta-analysis included only English language reports which may have restricted inclusion of some locally relevant articles. Some of the studies included in this review had small sample sizes such as 100. This meta-analysis represented only studies reported from five of nine regions (Amhara, Oromia, Tigray, Hararie and SNNP) and one of two administrative towns (Addis Ababa) of the country which could compromise the estimates of nutritional status at the national level.

## Conclusion

The prevalence of undernutrition, stunting, and underweight are moderately high while thinness (wasting) is at critical state among primary school children found to be greater than the under-five age group’s national average in 2016 EDHS. Therefore, this finding warrants the design of a school-based nutrition survey and implementation of programs to improve the nutritional status of primary school children in the country. In addition, special emphasis should be given for school aged children in the early stages of school years, creating programs targeting mothers who lack formal education, as well as preventing and early treating/deworming for parasitic infections. Moreover, the researchers try to conduct research on province/regions not yet studied for their school age children nutritional status to date.

## Data Availability

Minimal data can be accessed upon request from first author (MAA).

## Abbreviations

CI: Confidence Interval
EDHS: Ethiopian Demography and Health Survey
JBI: Joanna Briggs Institute
OR: Odds Ratio
PRISMA: Preferred Reporting Items for Systematic Review and Meta-Analysis
SD: Standard Deviation
SNNP: Southern Nation and Nationality and People
WHO: World Health Organization
