# Correction to: High burden of undernutrition among primary school-aged children and its determinant factors in Ethiopia; a systematic review and meta-analysis

**DOI:** 10.1186/s13052-020-00891-8

**Published:** 2020-09-09

**Authors:** Moges Agazhe Assemie, Alehegn Aderaw Alamneh, Daniel Bekele Ketema, Ali Mekonen Adem, Melaku Desta, Pammla Petrucka, Mekdes Marew Ambaw

**Affiliations:** 1 Biostatstics Unit, Department of Public Health, College of Health Science, Debre Markos University, P.O. Box: 269, Debre Markos, Ethiopia; 2 Department of Human Nutrition and Food Sciences, College of Health Sciences, Debre Markos University, Debre Markos, Ethiopia; 3 Biostatstics Unit, College of Health Science, Assossa University, Assossa, Ethiopia; 4 Department of Midwifery, College of Health Sciences, Debre Markos University, Debre Markos, Ethiopia; 5 College of Nursing, University of Saskatchewan, Saskatoon, Canada; 6 School of Life Sciences and Bioengineering, Nelson Mandela African Institute of Science and Technology, Arusha, Tanzania; 7 Department of Agricultural Economics, College of Agriculture and Resource Management, Dilla University, Dilla, Ethiopia

**Correction to: Ital J Pediatr 46, 118 (2020)**

**https://doi.org/10.1186/s13052-020-00881-w**

After publication of this article [1], it is noticed one of the author names is incorrect:

‘Melaku Desita’ should be corrected to ‘Melaku Desta’. The author name has been thus updated in this Correction.

The original article has also been updated.
